# Treatment of Hypertension Among Non-Cardiac Hospitalized Patients

**DOI:** 10.1007/s11886-022-01699-0

**Published:** 2022-05-07

**Authors:** Bhanu Chaganti, Richard A. Lange

**Affiliations:** Department of Cardiovascular Medicine, Texas Tech University Health Science Center El Paso, 4800 Alberta Avenue, El Paso, TX USA

**Keywords:** Hypertension, Inpatient HTN, Management, Non-cardiac Admissions

## Abstract

**Purpose of Review:**

This review provides a contemporary perspective and approach for the treatment of hypertension (HTN) among patients hospitalized for non-cardiac reasons.

**Recent Findings:**

Elevated blood pressure (BP) is a common dilemma encountered by physicians, but guidelines are lacking to assist providers in managing hospitalized patients with elevated BP. Inpatient HTN is common, and management remains challenging given the paucity of data and misperceptions among training and practicing physicians. The outcomes associated with intensifying BP treatment during hospitalization can be harmful, with little to no long-term benefits. Data also suggests that medication intensification at discharge is not associated with improved outpatient BP control.

**Summary:**

Routine inpatient HTN control in the absence of end-organ damage has not shown to be helpful and may have deleterious effects. Since routine use of intravenous antihypertensives in hospitalized non-cardiac patients has been shown to prolong inpatient stay without benefits, their routine use should be avoided for inpatient HTN control. Future large-scale trials measuring clinical outcomes during prolonged follow-up may help to identify specific circumstances where inpatient HTN control may be beneficial.

## Introduction

Although predominantly considered an outpatient condition, hypertension (HTN) is frequently observed and treated in the hospital setting where optimal practices for diagnosis and management are often uncertain. A systematic review of reports describing inpatient HTN prevalence found estimates ranging from 51 to 72% [1]. However, this percentage likely underestimates the prevalence since a considerable proportion of patients have HTN that is concealed or undetected unless 24-h blood pressure (BP) monitoring is performed during the hospitalization. Furthermore, about 28 to 38% of hospitalized patients with elevated BP have no prior diagnosis of HTN [2].

In the US guidelines, HTN in the outpatient setting is defined by an average systolic BP ≥ 130 mm Hg or diastolic BP ≥ 80 mm Hg [3]. Recognition of inpatient HTN can provide opportunities for diagnosis and treatment intensification during hospitalization and in the outpatient setting. However, determining which hospitalized patients with elevated BP have chronic HTN can be difficult in clinical practice, because there is currently no diagnostic standard, and numerous factors can impact BP measurements obtained in the hospital.

Despite strong evidence supporting the benefit of HTN management for prevention of cardiovascular disease in the primary care setting, there is a paucity of evidence supporting the value of strict control of BP in hospitalized patients. Health care providers regularly start or change medications to achieve BP control in hospitalized patients, even if the BP is only slightly elevated, with the belief that inpatient HTN control benefits patients [4]. In this review, available information regarding inpatient HTN management in non-cardiac hospitalized patients will be summarized and management recommendations offered.

## Basis for Treatment of Hypertension

In individuals with chronic HTN, BP lowering strategies have been shown to reduce the incidence of myocardial infarction, stroke, heart failure, and both cardiovascular and all-cause mortality. According to a large meta-analysis that included 68 randomized clinical trials conducted between 1966 and 2013, lowering systolic BP by 10 mm Hg in patients with chronic HTN reduces the risk of major cardiovascular disease events by 20%, coronary heart disease by 17%, stroke by 27%, heart failure by 28%, and all-cause mortality by 13% [5, 6].

A significant percentage of patients admitted to the hospital have undiagnosed HTN [7], and those with an established diagnosis of HTN are often not provided antihypertensive medications during their hospitalization [8]. When increased BP is observed in hospitalized patients, it is often attributed to anxiety, pain, or white coat HTN. Even among training resident physicians, surveys show they do not initiate treatment if BP is < 140/90 mm Hg [4].

Unless the hospitalized person has severely elevated BP, a clinical diagnosis of HTN is not considered based on the United Kingdom (UK), European, or American guidelines [9–11]. Accordingly, referral for community follow-up of these patients to determine the presence or absence of persistent HTN is poor. Unfortunately, studies show that patients with elevated BP recordings in the hospital frequently remain hypertensive in the community [2, 12–15]. Among patients without a history of HTN, the presence of elevated BP during hospitalization is associated with a higher risk of presenting with outpatient HTN at 1 month and 3 years after discharge [16, 17]. Thus, inpatient hospitalization provides an opportunity for identifying uncontrolled HTN and improving post-discharge BP control.

## Hypertension Treatment Guidelines

Major differences exist among the American [11], European [10], and Canadian [18] HTN guidelines in the BP thresholds recommended for pharmacological treatment of elevated BP. American guidelines recommend treatment with antihypertensives in patients > 20/10 mm Hg above BP goal, and individuals with BP > 130/80 mm Hg are considered to have HTN with recommendations to lower BP to < 130/80 mm Hg in all. In patients without macrovascular target organ damage or other cardiovascular risk factors, the Canadian guideline recommends initiation of pharmacological therapy when diastolic BP ≥ 100 mm Hg or systolic BP ≥ 160 mm Hg. In patients with macrovascular target organ damage or other cardiovascular disease risk factors, pharmacological treatment is recommended to be initiated when average diastolic BP ≥ 90 mm Hg or systolic BP ≥ 140 mm Hg; target BP levels are < 140/90 mm Hg [19]. The European HTN guideline recommends a BP treatment threshold of diastolic BP ≥ 90 mm Hg or systolic BP ≥ 140 mm Hg with ultimate treatment targets being systolic BP 120–129 mm Hg in those < 65 years old and 130–139 mm Hg in those 65 years and older [10].

None of the guidelines discusses the management of inpatient HTN in the absence of hypertensive emergencies or BP sensitive acute conditions such as acute myocardial infarction and acute ischemic stroke.

## Outcomes Associated with Treatment of Inpatient Hypertension

Despite the high prevalence of elevated BP among hospitalized non-cardiac patients, BP management guidelines are lacking for this population. A recent study of almost 23,000 adults hospitalized for non-cardiovascular diagnoses assessed the outcomes associated with acute HTN treatment (i.e., defined as administration of an intravenous antihypertensive medication or a new class of oral antihypertensive treatment) in patients who had a hypertensive BP (e.g., systolic BP > 140 mm Hg) recorded during their admission. Acute HTN treatment was associated with higher rates of subsequent acute kidney injury (10.3% vs 7.9%; *P* < 0.001) and myocardial injury (1.2% vs 0.6%; *P* = 0.003) than simply continuing medications prescribed before admission. When stratified by systolic BP (i.e., 140–159 mm Hg, 160–199 mm Hg, or > 200 mm Hg), there was no BP interval in which treated patients had better outcomes than untreated patients [20••]. Patients who received “as-needed” treatment with intravenous labetalol or hydralazine ordered at admission for elevated BP above an arbitrary threshold had longer hospitalizations than those for whom treatment was ordered but not administered: hospital length of stay was 10.6 ± 13.1 days (mean ± SD) for patients receiving hydralazine, 9.6 ± 11.1 days for those receiving labetalol, 13.5 ± 18.9 days for patients who received both drugs, and 6.5 ± 9.7 days for patients for whom drugs were ordered but not administered [21, 22•].

Initiating treatment for asymptomatic HTN in the emergency department is unnecessary when non-cardiac patients have follow-up. Furthermore, rapidly lowering BP in asymptomatic non-cardiac patients in the emergency department [23] or at hospital discharge may be harmful. Among older adults hospitalized for non-cardiac conditions, intensifying antihypertensive therapy at discharge did not reduce cardiovascular events or improve BP control at 1-year follow-up, but it was associated with an increased risk of readmission (hazard ratio [HR], 1.23; number needed to harm [NNH], 27) and serious adverse events (HR, 1.41; NNH, 63) within 30 days [24••].

The landmark Veterans Affairs Cooperative Trial [25] demonstrated the long-term benefits (including decrease in congestive heart failure, azotemia, transient ischemic attacks, cerebral hemorrhage, and myocardial infarction) of treating patients with chronic hypertensive urgency (elevated diastolic BP averaging 115–129 mm Hg). Importantly though, benefits occurred over a period of months to years…not hours or days. The time to the first adverse event in the placebo arm was 2 months, suggesting that those with BPs in the range of hypertensive urgency are unlikely to experience immediate (i.e., within hours) adverse events, even without strict BP control during hospitalization.

In fact, strict control of BP in the inpatient setting leads to adverse outcomes in many patients. In a series of 427 patients treated with intravenous nicardipine or nitroprusside for hypertensive emergency, 57% of patients experienced excessive reduction in BP (> 25% reduction in mean arterial pressure) within the first 30 min of treatment [26]. Sublingual nifedipine administered to inpatients with hypertensive urgencies has been associated with an increased incidence of ischemic neurovascular events, even after receiving small doses of medication that result in moderate BP reduction without resultant hypotension [27, 28]. Beta-blockers (intravenous labetalol), angiotensin-converting enzyme inhibitors (e.g., captopril), and clonidine have also been implicated in treatment-related adverse events [29, 30].

In contrast to the previously mentioned data, there are certain conditions where inpatient control of HTN is essential [31•]. In the International Stroke Trial (IST), persistent post-stroke HTN was associated with an increased risk of recurrent stroke within 14 days of presentation. Conversely, the IST also showed an ~ 18% increase in early death for every 10 mm Hg decrease in systolic BP below 150 mm Hg. Hence, carefully titrated inpatient BP control is essential in the management of stroke patients [32]. Additionally, American Heart Association guidelines for management of subarachnoid hemorrhage recommend that inpatient BP should be monitored and controlled to balance the risk of ischemic stroke with recurrent bleeding following the initial aneurysm rupture [33].

## Inpatient Hypertension Treatment Considerations

The decision to intensify antihypertensive therapy during hospital admission often results from the application of outpatient BP guidelines to the inpatient setting. However, many factors that are absent in the outpatient setting may contribute to transiently elevated inpatient BP including acute pain, stress, anxiety, and exposure to new drugs. Additionally, although few studies have examined the presence of a white coat effect for patients admitted to the hospital, recent research suggests that in-hospital BP in clinically stable patients is often higher than the BP measured at home after discharge [34]

Peri-operative HTN is common, and treatment should aim at preserving organ function, reducing complications, and improving outcomes. Because there are no management guidelines and no definitive treatment, the focus must be on tailoring therapy to the patient’s risk factors and comorbid morbidities as well as the clinical situation [35]. The treatment of acute elevations in BP (defined as an increase in systolic BP, diastolic BP, or mean arterial pressure by > 20% over baseline in the perioperative period) is without a uniform approach despite attempts to standardize the method to characterize intraoperative hemodynamics. One approach adopted by many providers is to base the treatment goal on the patient’s preoperative BP. A conservative target would be approximately 10% above baseline BP; unfortunately, no data supports the above — or any other target — goal [36].

## Long-Term Results of Inpatient Intensification of Treatment

In initiating or intensifying antihypertensive therapy for patients admitted to the hospital, clinicians may not be aware of important contextual factors, such as history of previous medical treatment, medication intolerance, barriers to adherence, and the patient’s long-term success at BP control [37]. Elevated inpatient BP is more common in patients with chronically elevated outpatient BP before admission. However, almost half of patients with elevated inpatient BP are normotensive prior to admission [36, 38]. In a study of 14,915 older adults (e.g., > 65 years of age) admitted to the hospital for common non-cardiac conditions, one in seven were discharged with intensified antihypertensive treatment even though half of the intensifications occurred in patients with previously well-controlled BP [22•]. No differences in rates of intensification were observed among patients least likely to benefit from tight BP control (i.e., limited life expectancy, dementia, or metastatic malignancy), nor in those most likely to benefit (i.e., history of myocardial infarction, cerebrovascular disease, or renal disease) suggesting that current treatment practices may focus on treating the BP “number” rather than treating the patient [39•].

Another study showed that antihypertensive medication regimen modifications during hospitalization of elderly patients were common, likely to be continued after hospital discharge, and were an independent risk factor for mortality 3 months post-discharge [40]. The initiation and intensification of antihypertensive medication is associated with a short-term increased risk of a serious fall injury in older adults. However, there does not seem to be a long-term increased risk for serious fall injuries associated with antihypertensive medication [41]. Clear communication remains paramount to minimizing the medication-related harms in the peri-hospitalization period; simply reconciling medications at discharge is not sufficient [40, 42].

## Conclusion

In non-cardiac hospitalized patients, outcome data do not support a benefit of treating acutely elevated BP; in fact, acutely lowering BP may be harmful. Accordingly, it is reasonable to limit the use of parenteral antihypertensive therapy in non-cardiac patients to situations in which acute target organ damage is suspected, rather than as a standing prn order targeted to elevated BP above a threshold level. Rather than focusing on the inpatient BP “numbers,” the goal should be to simplify and improve long-term HTN care. The decision to adjust antihypertensive medications in the inpatient setting should incorporate considerations of the patient’s likelihood of benefit in the context of the acute hospital situation and existing underlying chronic conditions.
